# Lipoteichoic Acid Induces Unique Inflammatory Responses when Compared to Other Toll-Like Receptor 2 Ligands

**DOI:** 10.1371/journal.pone.0005601

**Published:** 2009-05-19

**Authors:** Elizabeth M. Long, Brandie Millen, Paul Kubes, Stephen M. Robbins

**Affiliations:** 1 Departments of Oncology, Biochemistry and Molecular Biology and the Southern Alberta Cancer Research Institute, University of Calgary, Calgary, Canada; 2 Department of Biophysics and Physiology and The Institute of Infection, Immunity, and Inflammation, University of Calgary, Calgary, Canada; LMU University of Munich, Germany

## Abstract

Toll-like receptors (TLRs) recognize evolutionarily-conserved molecular patterns originating from invading microbes. In this study, we were interested in determining if microbial ligands, which use distinct TLR2-containing receptor complexes, represent unique signals to the cell and can thereby stimulate unique cellular responses. Using the TLR2 ligands, R-FSL1, S-FSL1, Pam2CSK4, Pam3CSK4, and lipoteichoic acid (LTA), we demonstrate that these ligands activate NF-κB and MAP Kinase pathways with ligand-specific differential kinetics in murine macrophages. Most strikingly, LTA stimulation of these pathways was substantially delayed when compared with the other TLR2 ligands. These kinetics differences were associated with a delay in the LTA-induced expression of a subset of genes as compared with another TLR2 ligand, R-FSL1. However, this did not translate to overall differences in gene expression patterns four hours following stimulation with different TLR2 ligands. We extended this study to evaluate the in vivo responses to distinct TLR2 ligands using a murine model of acute inflammation, which employs intravital microscopy to monitor leukocyte recruitment into the cremaster muscle. We found that, although R-FSL1, S-FSL1, Pam2CSK4, and Pam3CSK4 were all able to stimulate robust leukocyte recruitment in vivo, LTA remained functionally inert in this in vivo model. Therefore distinct TLR2 ligands elicit unique cellular responses, as evidenced by differences in the kinetic profiles of signaling and gene expression responses in vitro, as well as the physiologically relevant differences in the in vivo responses to these ligands.

## Introduction

Toll-like receptors (TLRs) are key components of the immune system's capacity to recognize infectious non-self and to mount a rapid and effective immune response [1]. They are type I transmembrane receptors, composed of an extracellular, leucine rich repeat (LRR), ligand-recognition motif, as well as a highly conserved, cytoplasmic, Toll/IL-1R (TIR), signaling-initiating domain [2], [3]. These receptors have evolved to recognize microbial molecules of bacterial, viral, and fungal origin that are comprised of molecular structures as diverse as proteins, lipopeptides, glycolipids, as well as nucleic acids. These ligands bind to either homo- or hetero-dimers of the TLR's, often in combination with different co-receptors [4].

TLR2, forming a heterodimer with either TLR1 or TLR6, is responsible for the recognition of bacterial lipoproteins and lipopeptides [5]. These ligands are derived from the bacterial cell membrane, where they are anchored via lipid chains attached to a polypeptide at a conserved N-terminal cysteine residue [6]. The number of fatty acids coupled to the N-terminus of the polypeptide is the crucial determinant in the ligand preference for specific TLR2 heterodimers. Triacylated lipoproteins are produced by most bacteria, with the exception of mycoplasma, and are recognized by TLR2/1 heterodimer complexes [7]. The third acyl chain in triacylated lipoproteins is attached via an amide bond to the N-terminal cysteine. This reaction depends on the presence of an N-acetyltransferase that is absent in mycoplasma and therefore these organisms produce only diacylated lipopeptides, which are recognized by TLR2/6 heterodimer complexes [8], [9]. In addition to these lipoproteins, TLR2 also recognizes and responds to the Gram-positive bacterial cell wall component, lipoteichoic acid (LTA) [10]. LTA is a diacylated, glycerophosphate polymer and as such, this ligand is recognized by a TLR2/6 heterodimer complex, presumably in a similar manner as the diacylated lipoproteins [11]. In short, the TLR2 heterodimer complexes allow for the accommodation of a structurally diverse ligand repertoire. We were particularly interested in whether the use of these distinct heterodimer complexes would translate to distinct, receptor complex-specific, responses to TLR2 ligands.

TLR1, TLR2, and TLR6 are all members of the TLR1-family of TLRs. These receptors share 66% sequence identity and are located in tandem on the same chromosome in mammals [12]. The sequence similarity between TLR1 and TLR6 is in part the result of gene conversion in a region encompassing the last four LRR motifs, the C-terminal cap, the transmembrane domain, and three quarters of the TIR domain [13]. These boundaries of gene conversion are tightly conserved across species, presumably as a result of an evolutionary pressure imparted by TLR1 and TLR6 shared functions, such as dimerization with TLR2 and the initiation of signaling responses. Despite the strict similarities between TLR1 and TLR6 there are some areas of divergence within both the LRR-motifs and the TIR domains. We hypothesized that the areas of free divergence within the TIR domain and at the C-terminus of the proteins may have evolved unique structure-function relationships, which could thereby impart ligand-specific cellular responses to TLR2-containing receptor complexes. Interestingly, employing distinct heterodimers is not the only way that TLR2 is able to extend its ligand repertoire, a forward genetics screen carried out by Beutler and colleagues, has revealed the requirement for the co-receptor, CD36, in order to mount a productive response to both LTA and the R-enantiomer of diacylated lipopeptides [14].

Based on the divergent sequences within the TLR2 binding partners TLR1 and TLR6, and the use of distinct, ligand-specific co-receptors, we questioned whether these differences would impart ligand-specific cellular responses to distinct TLR2 ligands. We evaluated the response of both immortalized and primary macrophages to different ligands that have been shown to signal through TLR2/1, TLR2/6, or TLR2/6/CD36. The TLR ligands used in this study were: the triacylated peptide, Pam3CSK3 (TLR2/1), the diacylated peptides, Pam2CSK4 (TLR2/6), S-FSL1 (TLR2/6), R-FSL1 (TLR2/6/CD36), and the diacylated glycerophosphate polymer, LTA (TLR2/6/CD36). We found that the different TLR2 ligands stimulated downstream signaling pathways with ligand-specific kinetic profiles in murine macrophages. Most strikingly, LTA consistently activated these pathways with a substantially delayed kinetic profile as compared with the other ligands. In addition, there was a delay in the rate of production of a subset of TLR-responsive transcripts in response to LTA (TLR2/6/CD36), as compared with R-FSL1 (TLR2/6/CD36). However, 4 hours following the initial ligand administration, the transcriptional profiles activated by each ligand were equivalent. Upon evaluating the in vivo pro-inflammatory potential of these ligands, we found that although Pam3CSK3 (TLR2/1), Pam2CSK4 (TLR2/6), S-FSL1 (TLR2/6), and R-FSL1 (TLR2/6/CD36), each induced robust leukocyte recruitment into the cremaster muscle, LTA (TLR2/6/CD36), was unable to cause any significant leukocyte recruitment. Therefore we have shown that there are differences in the interpretation of signals derived from ligands that signal through distinct TLR2-containing receptor complexes, as evaluated using both in vitro and in vivo assays.

## Materials and Methods

### Antibodies and Reagent

Pam3CSK4 and Pam2CSK4 were purchased from InvivoGen (San Diego, CA). R- and S-FSL1 were purchased from EMC microcollections (Tubingen, Germany). Ultrapure LTA was provided by Dr. T. Hartung (University of Konstanz, Konstanz, Germany). In this study we chose to use only synthesized di- or triacylated ligands as well as highly purified LTA, in order to avoid any of the prevailing issues of contamination. Antibodies for phospho-SAPK/JNK, total-SAPK/JNK, phospho-erk1/2, total-erk1/2, phospo-p38, phospho-c-jun, total-c-jun, phospho-ATF2, and IκBα were purchased from Cell Signaling Technologies (Beverly, MA). Anti-total p38 antibody was purchased from Santa Cruz Biotechnology (Santa Cruz, CA).

### Cell Culture and Western blotting

Western blots were performed as previously described [15]. Briefly, Raw 264.7 cells were plated in six-well tissue culture plates and allowed to grow to 85% confluence. The cells were treated with ligand for the indicated times, then lysed in Laemmili sample buffer and sonicated. The samples were then resolved using 10% SDS-PAGE. After electrophoresis, proteins were transferred to nitrocellulose membranes, which were then blocked by incubation in 5% BSA in TBS plus 0.05% Tween 20 (TBST). The membranes were then incubated at 4°C overnight in the primary antibody, washed for 40 minute in TBST, incubated for 1 h in secondary antibody, and washed for another 40 minute in TBST. The membranes were developed using an ECL substrate. For experiments using primary bone marrow derived macrophages, bone marrow was isolated from the femoral, tibial, and pelvic bones of wildtype C57BL/6 or TLR2-/- mice. These cells were cultured in DMEM + 10% FBS + penicillin/streptomycin/glutamine + 10% L929 cell conditioned media for 7 days, feeding the cells with fresh media on day 5. Treatments and Western blots were then performed as described above. Image J software (NIH) was used to quantify the Western blot signals.

### RT-PCR

RNA was isolate from relevant cells using Trizol® (Invitrogen) according to the manufacturer's protocol. Total RNA was treated with DNAase I and further purified by phenol extraction and precipitated with sodium acetate and ethanol. cDNA was prepared using superscript II (Invitrogen). For semi-quantitative RT-PCR the cDNA was amplified using the following gene-specific primers for TNFα, 5′TTGACCTCAGCGGCTGAGTTG3′ and 5′CCTGTAGCCCACGTCGTAGC3′. The PCR products were prepared with 10× DNA loading dye and run on a 2% TAE-agarose gel. Pictures were taken using the Gel Doc 2000 system (BioRad), with the saturated pixels function engaged so as to avoid overexposing the image. Image J software (NIH) was used to quantifiy the bands on the gel. In the case of the SABiosciences arrays, cDNA was diluted with water according to the manufacture's instructions and further mixed with the PCR mastermix supplied by the manufacture. 100 μL of this final mixture was added to each well of the array, (RT^2^Profiler PCR Array, Mouse Inflammatory Cytokines and Receptors), and run in the ABI7000 RT-PCR machine using the following protocol, 95°C 10 minutes, 40 cycles of 15 seconds 95°C, and 1 minute 60°C. The normalized threshold cycle (C_t_) value for each gene, (ΔC_t_), was determined by subtracting the C_t_ value of the housekeeping gene, actin, from the C_t_ value of the gene of interest. The change in the C_t_ value between control and treated samples for each gene of interest, (ΔΔC_t_), was then calculated and the fold change was determined using the following formula, 2^∧^(−ΔΔC_t_).

### Mice

Experiments were performed using male C57/B6 wildtype mice purchased from The Jackson Laboratory (Bar Harbor, ME) or TLR2-/- provided by Prof. Shizuo Akira (Osaka University, Japan). These mice were maintained in a pathogen-free facility at the University of Calgary's Animal Resource Center. At the time of use, mice weighed between 20 and 30 g and were 6–10 wk old. Experimental animal protocols performed in this study were approved by the University of Calgary Animal Care Committee and met the guidelines of the Canadian Council for Animal Care.

### In vivo evaluation of TLR2 ligands

Wildtype C57/B6 mice were given an intrascrotal injection of 150 μL of saline alone, or saline containing, TNFα (20 ng/g), LPS (10 ng/g), LTA (5 ng/g or 25 ng/g), Pam3CSK4 (5 ng/g), Pam2CSK4 (5 ng/g), R-FSL1 (5 ng/g), or S-FSL1 (5 ng/g). Leukocyte adhesion and emigration were measured in the cremasteric venules using intravital microscopy, 4.5 h following the injection. Intravital microscopy was performed on male mice, which were anaesthetized with an intraperitoneal injection of a mixture of 10 mg/kg xylazine (Bayer, Animal Health, Toronto, ON, Canada) and 200 mg/kg ketamine hydrochloride (Rogar/STB, Montreal, QC, Canada). For all protocols, the left jugular vein was cannulated to administer additional anaesthetic or drugs when necessary. The mouse cremaster muscle preparation was used to study the behaviour of leukocytes in the microcirculation and adjacent muscle tissue as previously described [16]. Briefly, an incision was made in the scrotal skin to expose the left cremaster muscle, which was then carefully dissected free of the associated fascia. The cremaster muscle was cut longitudinally with a cautery. The testicle and the epididymis were separated from the underlying muscle and were moved into the abdominal cavity. The muscle was then held flat on an optically clear viewing pedestal and was secured along the edges with 4-0 suture. The exposed tissue was superfused with 37°C-warmed bicarbonate-buffered saline (pH 7.4). An upright microscope (Mikron; Carl Zeiss Canada, Don Mills, ON, Canada) with a 20× objective lens (Zeiss LD Plan-NEOFLUAR) and a 10× eyepiece was used to examine the cremasteric microcirculation. A video camera (XR/MEGA10-AM Panasonic, Osaka, Japan) was used to project the images onto a monitor, and the images were recorded for playback analysis using a DVD recorder. Three to five single unbranched cremasteric venules (25 to 40 μm in diameter) were selected and the average number of adherent and emigrated leukocytes was determined offline during playback analysis. A leukocyte was considered to be adherent if it remained stationary for at least 30 seconds, and total leukocyte adhesion was quantified as the number of adherent cells within a 100-μm length of venule. Leukocyte emigration was defined as the number of cells in the extravascular space within the field of view, a 550 μm^2^ area, adjacent to the observed venule. Only cells adjacent to, and clearly outside, the vessel under study were counted as emigrated.

### Cremaster Histology

Wildtype male C57/B6 mice were given an intrascrotal injection of saline, (150 μl), or saline containing various proinflammatory ligands, as described in the results. Four hours following ligand administration the cremaster muscle was exteriorized, cut off, and fixed in 10% formalin. The cremaster tissue was embedded in paraffin and 5 μm sections were cut and stained with haematoxylin and eosin. The numbers of lymphocytes, monocytes, and neutrophils in the post capillary venules were counted and differential percentages were scored.

### Statistical analysis

The results are expressed as means ± SEM. A one-way analysis of variance was applied for multiple comparisons.

## Results

### Individual TLR2 ligands activate signaling pathways with unique kinetic signatures in murine macrophages, in a TLR2-dependent manner

**Figure 1:**
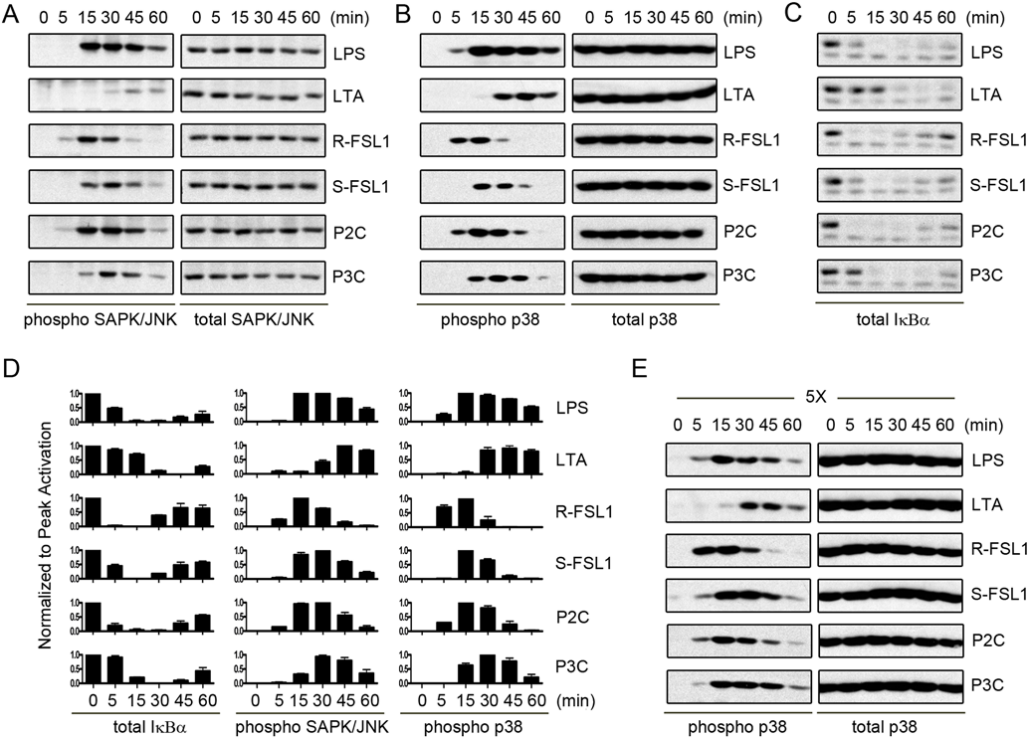
Kinetic profiles of signaling pathway activation in Raw 264.7 cells, in response to TLR2 ligands. Raw 264.7 cells treated with LPS (100 ng/mL), LTA (1 μg/mL), R-FSL-1 (100 ng/mL), S-FSL-1 (100 ng/mL), Pam2CSK4 (100 ng/mL), Pam3CSK4 (100 ng/mL) for 0, 5, 15, 30,45, or 60 minutes were used to perform Western Blot analysis of A) phospho-SAPK/JNK activation signal and total-SAPK/JNK protein amounts, and B) Phospho-p38 activation signal and total-p38 protein amounts and C) total IκBα protein amounts. In D) the normalized levels of total IκBα protein, phospho-SAPK/JNK activation signal, or phospho-p38 activation signal are shown for n = 3 experiments. Western blot band intensities were quantified using Image J software (NIH) and all signals were background subtracted and then normalized to peak activation levels. Therefore peak activation was given a value of 1. In E) Raw 264.7 cells, stimulated with 5-fold the initial ligand concentrations, LPS (500 ng/mL), LTA (5 μg/mL), R-FSL-1 (500 ng/mL), S-FSL-1 (500 ng/mL), Pam2CSK4 (500 ng/mL), Pam3CSK4 (500 ng/mL) for 0, 5, 15, 30,45, or 60 minutes were used to perform Western blot analysis of phospho-p38 activation signal and total-p38 protein amounts. Results are representative of n = 3 independent experiments.

To examine whether different TLR2 ligands activate cells in ligand-specific ways, we first stimulated the murine macrophage/monocyte cell line, Raw 264.7, for 0, 5, 15, 30, 45, or 60 minutes with each of the TLR2 ligands: LTA (TLR2/6/CD36), R-FSL1 (TLR2/6/CD36), S-FSL1 (TLR2/6), Pam2CSK4 (TLR2/6), or Pam3CSK4 (TLR2/1). Whole-cell lysates from these cells were analysed by Western blotting and it was observed that the MAP Kinase as well as the NF-κB signaling pathways, which are well known targets of TLR activation, were differentially activated by the different TLR2 ligands (Fig. 1). The activation of the MAP Kinase pathways was determined by blotting for phospho-SAPK/JNK (Fig. 1A) and phospho-p38 (Fig. 1B), and the degradation of IκBα was indicative of the activation of the NF-κB pathway (Fig. 1C). The ERK1/2 MAP Kinase pathway also showed similar activation kinetics to the SAPK/JNK and p38 pathways (data not shown). All of these pathways were rapidly and robustly activated when cells were treated with R-FSL1, S-FSL1, Pam2CSK4 and Pam3CSK4, although there were reproducible differences in the activation kinetics between these ligands. R-FSL1 consistently activated all signaling pathways very quickly; by 5 minutes the phospho-MAP Kinase pathway activation markers and the degradation of IκBα were apparent and these signals generally subsided by 30–45 minutes. Pam2CSK4 followed this trend closely but tended to be slightly delayed as compared with R-FSL1. In contrast, Pam3CSK4 and S-FSL1 did not lead to any visible activation of the MAP Kinase or NF-κB pathways until the 15 minute timepoint and this signal generally remained until the 45–60 minute timepoint. Although these ligands were similar, Pam3CSK4 was slightly delayed when compared with S-FSL1. However, the biggest difference in the kinetics of activation between the different TLR2 ligands was observed when LTA was compared with the other TLR2 ligands. LTA treatment did not activate the MAP Kinase or NF-κB pathways until the 30 minute timepoint, and these signals began to subside by the 60 minute timepoint. Figure 1D shows the relative quantification of the kinetics of activation of these pathways for three independent experiments. The observed differences in the kinetic profiles between the distinct TLR2 ligands were not the result of differences in the effective concentrations of the ligands used. When ligand concentrations were increased by five-fold, the relative kinetic profiles were exactly the same as that observed using our initial concentration, as shown for p38 MAP Kinase (Fig. 1E) and SAPK/JNK (data not shown). Although the overall kinetic profiles were the same, the signal strength of activation of these pathways was more robust when the higher ligand concentration was used. Therefore in the remaining studies we chose to continue using the initial ligand concentrations, as this resulted in consistent cellular stimulation, which was not altered upon increasing the ligand concentration, and are consistent with what has been used in the literature.

**Figure 2:**
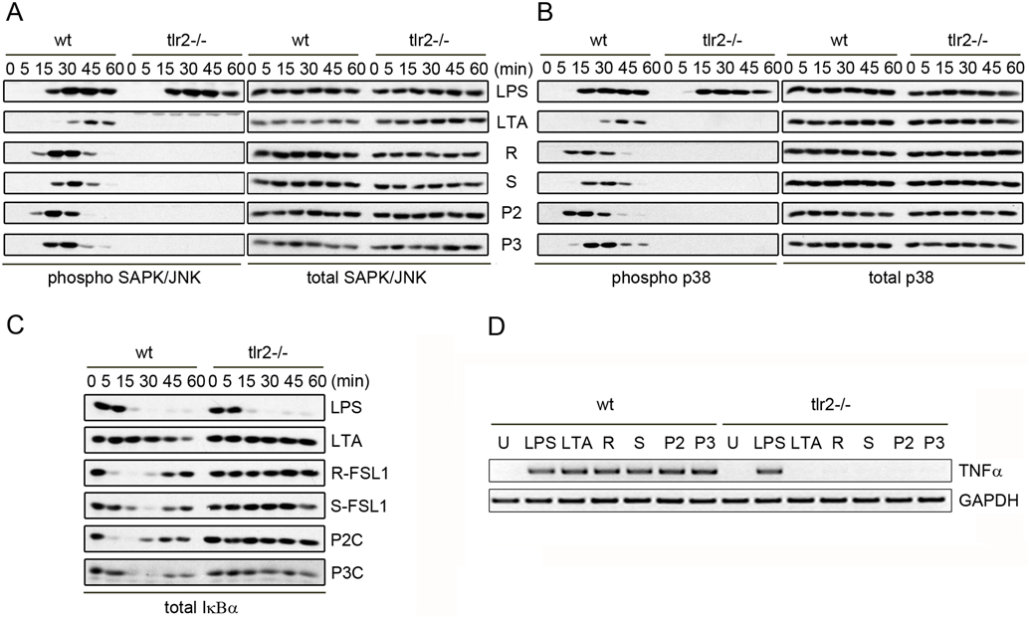
Kinetic profiles of signaling pathway activation in response to TLR2 ligands in BMDMs. Primary bone marrow derived macrophages (BMDMs), isolated from either wildtype or TLR2-/- mice, treated with LPS (100 ng/mL), LTA (1 μg/mL), R-FSL-1 (100 ng/mL, R), S-FSL-1 (100 ng/mL, S), Pam2CSK4 (100 ng/mL, P2), Pam3CSK4 (100 ng/mL, P3) for 0, 5, 15, 30,45, or 60 minutes were used to perform Western blot analysis of A) phospho-SAPK/JNK activation signal and total-SAPK/JNK protein amounts, B) Phospho-p38 activation signal and total-p38 protein amounts, and C) total IκBα protein amounts. In D) RT-PCR was performed on mRNA isolated from wildtype or TLR2-/- bone marrow derived macrophage treated with each ligand for 4 hours and the TLR2-dependence of cell activation leading to TNFα mRNA transcription is shown. All results are representative of n = 3 independent experiments.

To ensure that the difference in activation kinetics we observed in the Raw 264.7 cells was not a cell-line specific phenomenon, similar experiments were performed using bone marrow derived macrophages, isolated and derived from the bone marrow of wildtype C57/B6 mice. When these macrophages were fully differentiated and treated with the TLR2 ligands: LTA, R-FSL1, S-FSL1, Pam2CSK4, or Pam3CSK4, once again there were ligand-specific activation kinetics of the p38, SAPK/JNK, and NF-κB pathways, which precisely matched those observed using the immortalized Raw 264.7 cells (Fig. 2A–C). These primary bone marrow derived macrophages were also used to ensure that the responses to these ligands were TLR2-specific. Bone marrow from TLR2-/- mice was used to differentiate into macrophages and these cells were treated with the different TLR2 ligands alongside the macrophages derived from wildtype bone marrow. When these TLR2-/- macrophages were evaluated with respect to the activation of MAP Kinase or NF-κB pathways, these ligands were unable to activate any pathways in these cells, indicating the TLR2-dependence of all these responses. As expected the TLR4 ligand, LPS was able to active wildtype and TLR2-/- cells (Fig. 2A–C). To further verify that these ligands only stimulate responses in wildtype and not TLR2-/- cells, we treated bone marrow derived macrophages isolated from both strains of mice, with the TLR2 ligands: LTA, R-FSL1, S-FSL1, Pam2CSK4, or Pam3CSK4 for 4 hours. These cells were then lysed and RNA was extracted for RT-PCR-based evaluation of pro-inflammatory responses. The mRNA transcript for TNFα was substantially upregulated in wildtype cells treated with each of the TLR2 ligands, or when LPS was included as a positive control. However, the TLR2-/- cells did not contain an increased amount of the TNFα transcript when cells were treated with the TLR2 ligands. LPS was still able to stimulate TNFα transcript production in these TLR2-/- cells (Fig. 2D).

### The difference in the activation kinetics of the MAPK Kinase pathways translates to a difference in the activation kinetics of downstream transcription factors

**Figure 3:**
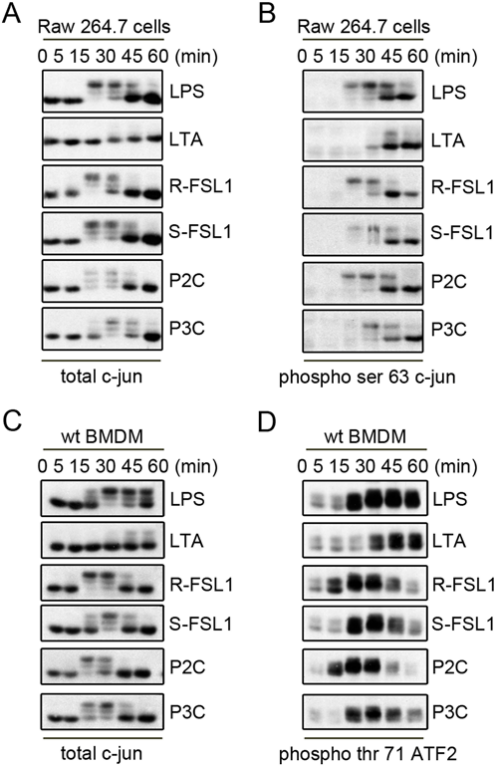
Kinetic profiles of transcription factor activation in response to TLR2 ligands. Raw 264.7 cells were treated with LPS (100 ng/mL), LTA (1 μg/mL), R-FSL-1 (100 ng/mL), S-FSL-1 (100 ng/mL), Pam2CSK4 (100 ng/mL), Pam3CSK4 (100 ng/mL) for 0, 5, 15, 30,45, or 60 minutes and lysates were used for Western blot analysis of A) total c-jun and B) phospho-Ser63 c-jun. Wildtype bone marrow derived macrophage were treated in a similar way as the Raw 264.7 cells in A and B and lysates were used for Western blot analysis of C) total c-jun and D) phospho-Thr71 ATF2. All results are representative of n = 3 independent experiments.

Since we observed differences in the activation kinetics of the MAP Kinases, we wanted to evaluate whether this translated to differences in the activation of their downstream transcription factors. We evaluated the activation of the transcription factor, c-jun, by its phosphorylation on an activating residue, Ser63, as well as the electrophoretic shift caused by the phospho-modification of the protein when Western blots were probed with a total anti-c-jun antibody (Fig. 3A and B). Consistent with our earlier observations, there were reproducible, albeit subtle, kinetic differences in the phosphorylation and presumed activation of c-jun when Raw 264.7 cells were treated with, R-FSL1, S-FSL1, Pam2CSK4, or Pam3CSK4. More dramatically, the activation of c-jun by LTA was much weaker than that observed with any of the other TLR2 ligands and once again this activation was not apparent until 30–45 minutes, which was substantially delayed when compared with the other TLR2 ligands. In wildtype bone marrow derived macrophages, the kinetics of c-jun activation also followed similar kinetic profiles, as judged by the electorphoretic shift of total c-jun (Fig. 3C). Another transcription factor that is downstream of the initiating MAP Kinases, ATF2, also showed differential activation kinetics in wildtype bone marrow derived macrophages, as measured by Thr71 phosphorylation of this protein (Fig. 3D).

### The difference in activation kinetics of the MAP Kinase and NF-κB pathways downstream of different TLR2 ligands does not translate to different gene expression profiles

**Figure 4:**
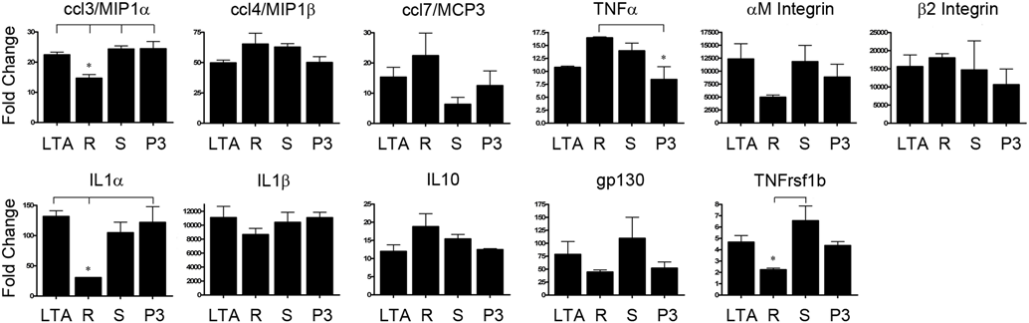
Transcriptional profile in response to distinct TLR2 ligands. Raw 264.7 cells were treated for 4 hours with LTA (1 μg/mL), R-FSL-1 (100 ng/mL, R), S-FSL-1 (100 ng/mL, S), or Pam3CSK4 (100 ng/mL, P3), mRNA was extracted and used to perform arrays using the RT^2^Profiler PCR Array, Mouse Inflammatory Cytokines and Receptors (SABiosciences). Genes that were consistently upregulated greater than 2 fold over n = 3 independent experiments are shown. (* p<0.05).

Considering that ATF2 and c-jun, both components of the AP-1 transcription factor complex, and the primary innate immune regulator, IκBα, are each activated with differential kinetics by the TLR2 ligands we tested, we wanted to evaluate the possible influence that these differential signaling kinetics would have on the transcriptional response of macrophages to the different TLR2 ligands. We employed the real-time RT^2^-Profiler™ PCR Array System from SABiosciences, to evaluate the transcriptional response of known TLR response genes. Raw 264.7 cells were treated for four hours with either: LTA, R-FSL1, S-FSL1, or Pam3CSK4, and subsequently analysed for the upregulation of pro-inflammatory transcripts. Judged by these real-time RT-PCR arrays, all of the genes that were upregulated following treatment with each of the TLR2 ligands were the same (Fig. 4). However, there were some differences in the degree to which different genes transcripts were upregulated. Of the 11 genes that were shown to be consistently upregulated by the TLR2 ligands, three genes; ccl3/MIP1α, IL1α, and the TNF receptor superfamily member 1b, were all upregulated to a lesser degree 4 hours following stimulation with R-FSL1 as compared with the other TLR2 ligands.

### The transcriptional response of some genes is delayed in response to LTA versus R-FSL1

**Figure 5:**
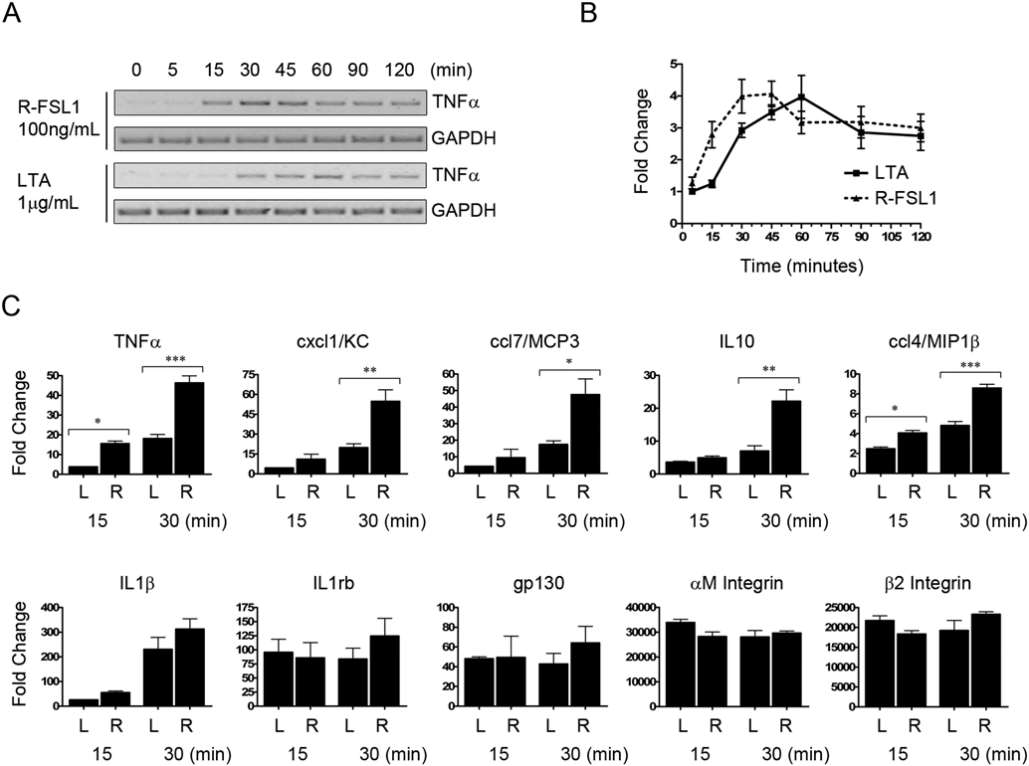
Kinetic profiles of transcriptional responses to different TLR2 ligands. Raw 264.7 cells were treated for 0, 5, 15, 30, 45, or 60 minutes with either R-FSL1 (100 ng/mL, R) or LTA (1 μg/mL, L). The transcriptional upregulation of the TNFα gene was monitored using A) RT-PCR and the results of n = 3 independent experiments of semi-quantitative PCR were analyzed using Image J software (NIH) and are presented in B). In C), the kinetic profile of the transcriptional response to other TLR2-responsive genes was analyzed using the RT^2^Profiler PCR Array, Mouse Inflammatory Cytokines and Receptors (SABiosciences). Genes that were consistently upregulated greater than 2 fold over n = 3 independent experiments at these timepoints are shown. (* p<0.05, ** p<0.01, and *** p<0.001).

To further evaluate whether different TLR2 ligands are able to influence the transcriptional response of murine macrophages in distinct ways, we examined the kinetics of the transcriptional response to these ligands in Raw 264.7 cells. Since these ligands can cause distinct kinetics of activation of downstream signaling pathways, we chose to evaluate the ligands that showed the greatest differences in these assays, LTA and R-FSL1. Raw 264.7 cells were treated for 0, 5, 15, 30, 45, 60, 90, and 120 minutes with either LTA or R-FSL1, and RNA was extracted from these cells and evaluated for the presence of the TNFα transcript. In cells treated with R-FSL1, there was a rapid up-regulation of TNFα mRNA transcripts, as demonstrated by RT-PCR, with cDNA synthesis apparent from these samples as early as 15 minutes following stimulation. However in cells that had been treated with LTA, evidence of the TNFα transcripts was not detectable until 30 minutes (Fig. 5A–B). This is consistent with the kinetics of activation of the signaling pathways being delayed upon treatment of cells with LTA as compared with the other TLR2 ligands tested. To extend these results to other inflammatory genes we once again employed the real-time RT^2^-Profiler^TM^ PCR Arrays. RNA extracted from Raw 264.7 cells, which had been treated with LTA or R-FSL1 for 15 or 30 minutes, was used to perform the arrays (Fig. 5C). We found both R-FSL1 and LTA ultimately led to the up-regulation of the same genes; however, the transcriptional response of some genes in response to LTA was delayed. TNFα, cxcl1/KC, ccl7/MCP3, IL10, and ccl4/MIP1β each exhibited this delayed transcriptional response to LTA as compared with R-FSL1. In contrast, the other gene transcripts that we found to be up-regulated at either the 15 or 30 minute timepoint were equally responsive to both R-FSL1 and LTA. IL1β, IL10 receptor β, gp130, αM Integrin, and β2 Integrin gene transcription was all up-regulated to an equivalent extent and with similar kinetic profiles upon treatment with either R-FSL1 or LTA.

### R-FSL1, S-FSL1, Pam2CSK4, and Pam3CSK4 each induce robust leukocyte recruitment in vivo, in contrast, LTA is unable to induce leukocyte recruitment in vivo

**Figure 6:**
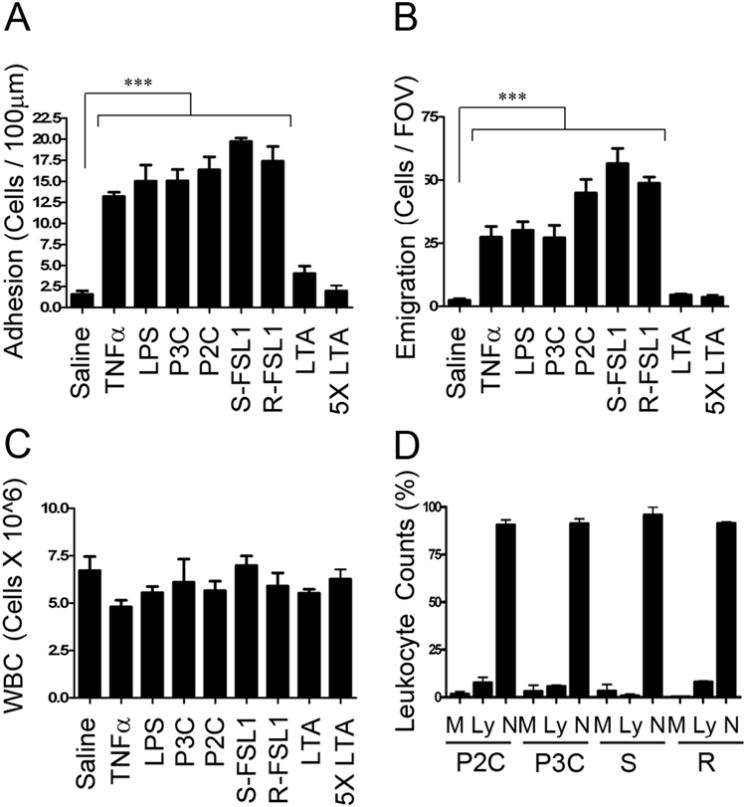
Evaluation of the in vivo pro-inflammatory capacity of distinct TLR2 ligands. Wildtype, male, C57/B6 mice were given intrascrotal injections of either saline alone, or saline containing TNFα (20 ng/g), LPS (10 ng/g), Pam3CSK4 (5 ng/g), Pam2CSK4 (5 ng/g), S-FSL1 (5 ng/g, S), R-FSL1 (5 ng/g, R), LTA (5 or 25 ng/g). After 4 hours the cremaster muscle was exteriorized and the degree of leukocyte A) adhesion and B) emigration was measured. In C) total circulating leukocytes were counted in the blood. In D) the relative percentages of lymphocytes (Ly), monocytes (M), and neutrophils (N) within the post-capillary venules were determined from haematoxylin and eosin stained cremaster tissue sections. All results are representative of n≥3 independent experiments. (*** p<0.001).

Following from our observations in vitro we were interested in evaluating the response to distinct TLR2 ligands in an in vivo setting. Our group has previously investigated the in vivo responses to the TLR2 ligand, LTA, and we found that LTA is unable to induce leukocyte recruitment when administered in vivo [17]. These studies showed that following an intrascrotal injection of highly purified LTA, there is no significant increase in the number of leukocytes rolling along the venules, adhering to the venules, or emigrating into the tissue of the murine cremaster muscle. In our current study we evaluated the inflammatory potential of other known TLR2 ligands to determine if the absence of leukocyte recruitment observed upon in vivo stimulation with LTA was a characteristic of all TLR2 ligands, or phenomenon that is unique to LTA. Under control conditions, 4 hours following an intrascrotal injection of saline, there were very few cells adhering to the post-capillary venules (Fig. 6A) or that had emigrated into the tissue (Fig. 6B) of the murine cremaster muscle. Upon intrascrotal injection of the positive controls, LPS or TNFα, there were approximately 12–15 leukocytes adherent (Fig. 6A) within the post-capillary venules and 30 leukocytes that had emigrated (Fig. 6B) into the tissue, 4 hours post-stimulation. In contrast, the intrascrotal administration of the TLR2 ligands yielded mixed results. Whereas LTA did not induce any significant leukocyte adhesion (Fig. 6A) or emigration (Fig. 6B), the other TLR2 ligands that were tested: R-FSL1, S-FSL1, Pam2CSK4, and Pam3CSK4, each induced on average between 15–19 cells to adhere (Fig. 6A) within the venules and 30–70 cells to emigrate (Fig. 6B) into the tissue. To ensure that the lack of a response to LTA was not simply due to the fact that LTA may require a higher active concentration, 5× the initial concentration of LTA was administered by intrascrotal injection and once again no significant adhesion (Fig. 6A) nor emigration (Fig. 6B) was observed within the cremaster muscle. These results are in agreement with our previous studies with LTA [17]. LTA is unique among the TLR2 ligands tested in the cremaster model, in that this ligand appears to be functionally inert with respect to leukocyte recruitment, whereas the other TLR2 ligands were able to induce robust leukocyte recruitment. Since LTA was not leading to the recruitment of cells into the tissue we also recorded the circulating leukocyte counts in order to ensure that LTA was not causing leukopenia. Figure 6C shows that none of the ligand treatments caused a reduction in the circulating leukocyte numbers. Finally, cremaster tissue sections were stained with haematoxylin and eosin in order to histologically examine the types of leukocytes that were recruited into the vasculature of the murine cremaster muscle. The relative percentages of lymphocytes, monocytes, and neutrophils, as a percentage of total leukocytes within post-capillary venules, are shown in Figure 6D. The vast majority (>85%) of leukocytes within these venules were neutrophils, and there were no significant differences between the profiles of cells recruited into the post-capillary venules in response to the different TLR2 ligands (Pam2CSK4, Pam3CSK4, S-FSL1, or R-FSL1). Lipoteichoic acid did not cause any cell recruitment into the cremaster muscle and therefore the relative percentages of cells within the venules are not shown.

## Discussion

Our studies have revealed that although the TLR2 ligands, R-FSL1, S-FSL1, Pam3CSK4, Pam2CSK4, and LTA each activate typical TLR-dependent pathways in vitro, the kinetics of this activation is substantially delayed in response to LTA as compared with the other TLR2 ligands. LTA is also unable to stimulate leukocyte recruitment in vivo; whereas, the other TLR2 ligands are all able to provoke robust leukocyte recruitment into the murine cremaster muscle, following intrascrotal injection of the ligands. Together these distinct responses represent substantial, physiologically relevant differences in the interpretation of the signal initiated by different TLR2 ligands.

Similar to what has already been extensively characterized in the literature; we show that each of the TLR2 ligands is able to activate the downstream MAP Kinase and NF-κB pathways in vitro in murine macrophages. However, when the kinetic profiles of activation of these pathways were considered, we found substantial differences between the kinetics of activation induced by each TLR2 ligand. This was most apparent when the activation kinetics induced by LTA was compared with the other TLR2 ligands tested. LTA was dramatically delayed in activation of both the MAP Kinase pathways as well as the NF-κB pathways. Although these kinetic differences did translate to the level of downstream transcription factors, they did not affect the overall gene expression patterns activated downstream of the different TLR2 ligands 4 hours following cellular stimulation with each ligand. This is consistent with a recent study which used extensive microarray analysis and also showed that distinct TLR2 ligands did not induce unique gene expression profiles [18]. However, when we evaluated the kinetics of the transcriptional activation, we found that although LTA and R-FSL1 activated the same transcriptional response, treatment with LTA did not result in the transcription of many inflammation-associated genes nearly as quickly as did R-FSL1. This is in line with our observation that the kinetics of activation of upstream signaling pathways is delayed upon cellular stimulation with LTA as compared with the other TLR2 ligands. Notably, not all of the upregulated transcripts were delayed upon treatment with LTA versus R-FSL1. Some genes, IL10 receptor β, gp130, αM Integrin, and β2 Integrin, showed robust transcription at the 15 minute timepoint, which is well before the observable activation of the upstream signaling pathways by LTA. It is possible that the limited resolution of Western blots cannot fully appreciate the interpretation of local activation of pathways within the cell. Therefore there may be threshold activation of some signals that are important to the cell, well before they become observable using Western blot analysis. Clearly, the cellular mechanisms that regulate gene expression are multifaceted and this may explain our observation that genes induced downstream of LTA fall into two distinct categories; those that are delayed in their response as compared with R-FSL1 and those that are induced with comparable kinetics to R-FSL1. For example, it is possible that the regulation of chromatin dynamics, and thereby the accessibility of the DNA to transcriptional machinery, is impaired at specific loci in response to LTA as compared with R-FSL1. This mechanism of transcriptional specificity has been demonstrated to help establish the unique transcriptional responses downstream of distinct TLRs or to allow sequential gene expression profiles to be initiated downstream of TLR4 [19], [20]. In a similar way, the activation of some transcriptional programs downstream of TLR4 require strong, sustained activation of NFκΒ pathways in order to feed forward and activate other transcription factors, such as C/EBPδ, which further enhance the transcription of a subset of genes [21]. Considering these dynamic regulatory mechanisms, it is reasonable to speculate that the differences in the kinetics of activation of downstream pathways, activated by the distinct TLR2-complex containing ligands, would influence the overall cellular responses to these ligands. The complementary in vivo observations we present in this study may be the consequence of some of these distinct regulatory responses working within the tissue to establish a productive inflammatory response to R-FSL1, S-FSL1, Pam3CSK4, and Pam2CSK4; whereas the response to LTA is ultimately unproductive in vivo.

Contrary to our in vivo results, there are reports that LTA causes acute pulmonary inflammation in vivo [22]. We have also observed that intratracheal administration of LTA to mice resulted in substantial neutrophil infiltration into the lung (data not shown). The striking difference between our results in the cremaster and these studies may be reflective of cell type specific, and thus organ specific, immune responses.

We anticipate that the differences in the kinetic signaling responses to the TLR2 ligands may be as a result of differences in the heterodimer selection or the use of distinct co-receptors by individual ligands. To date, there have not been any TLR1- or TLR6-specific adaptor proteins identified, which would impart TLR1- or TLR6-specific signaling responses. However the unique in vitro and in vivo responses to distinct ligands that we report in this study may support this as a legitimate avenue of investigation. In terms of co-receptors, TLR2 has been shown to require CD36 to respond to diacylated ligands. CD36 has been shown to activate Src-family Kinases [23], and this may impact the overall signaling milieu and eventual response to these ligands. However, as both R-FSL1 and LTA have been shown to employ this co-receptor this could not explain the differential response to LTA versus R-FSL1 that we observed in this study. Recently, integrin β3 was shown to be involved in the response to both lipopeptides and LTA [24]. This integrin forms part of the TLR2-containing ligand recognition complex and is essential for proinflammatory cytokine production in response to these ligands. It is possible that other distinct, heretofore unidentified co-receptors may be responsible for the distinct responses to the different TLR2 ligands we observed in this study.

In addition to the distinct heterodimer and co-receptor involvement in ligand recognition, the cellular trafficking of these complexes following ligand stimulation may influence the final cellular response. The growth factor signaling field has shown that for efficient signaling to occur, signals must be propagated from distinct cellular locations. For example, an endocytosis-defective dynamin mutant that blocks internalization of the EGF receptor has been found to prevent maximal EGF-induced ERK activation [25]. Similarly within the TLR field, Medzithov and colleagues have recently shown that TLR4 driven, Myd88/Mal-dependent and TRIF/TRAM-dependent signaling pathways are initiated from the plasma membrane and endosomal compartments, respectively [26]. In the case of TLR2, it has been shown that inhibiting internalization of LTA can actually increase the in vitro cellular response to ligand stimulation in murine macrophages. Internalized LTA co-localizes with ER, Golgi, and endosomal markers, in direct contrast to the other TLR2 ligands, Pam3CSK4 and FSL-1, which are found in endocytic vesicles. Additionally, or as a direct result of these differences, LTA is internalized much more quickly than these other TLR2 ligands [27]. Taken together, this evidence may indicate that the molecular structure of LTA dictates a vastly different internalization program than the other TLR2 ligands, which may impede the ability of this ligand to promote robust signaling and a productive immune response.

As discussed, when macrophages were treated with LTA, the activation of the MAP Kinase and NF-κB pathways was delayed by approximately 15 minutes when compared with the activation profiles of the other TLR2 ligands. We suspected that this difference in activation kinetics would translate to greater differences in the cellular response to these ligands. As a precedent, the differential response of PC12 cells to NGF, which causes neuronal differentiation, versus EGF, which yields a weak proliferative response, has been attributed to sustained versus transient activation of the ERK MAP Kinase pathway [28]. Within the TLR4 field, it is well known that early activation of downstream signaling pathways is dependent on MyD88/Mal-dependent signaling from the plasma membrane and that these pathways demonstrate sustained activity on the basis of continued stimulation through the TRIF/TRAM-dependent signaling complex at the endosome. Recently, it has been shown that in the absence of CD14, TLR4 retains signaling abilities but only through the MyD88/Mal-dependent pathway [29]. Moreover, specific ligands have been shown to utilize this paradigm in a functionally relevant manner. Rough LPS, (poorly glycosylated), does not require CD14, and smooth LPS, (highly glycosylated), does require CD14 to activate cells in a TLR4 dependent manner [29]. The differences in the pathways activated downstream of these ligands is of paramount importance to the types of genes which are upregulated in response to these stimuli. TLR4 has also been shown to activate only the TRIF/TRAM-dependent pathway downstream of the vesicular stomatitis virus glycoprotein G. This activation is principally dependent on TRAM and allows for only a type I interferon response in the absence of a MyD88/Mal-dependent activation of NFκB [30]. The specific intricacies of each ligand notwithstanding, the differential activation is manifest as a differential kinetics of activation, as MyD88/Mal-dependent versus TRIF/TRAM-dependent pathways tend to proceed quickly from the plasma membrane and delayed from the endosome, respectively. These findings represent a model whereby both the supramolecular TLR4-containing complex, in cooperation with the nature of the ligand, determine how the receptor will signal and ultimately direct the cellular and organismal response. Although we did not observe any lasting, distinct transcriptional responses to the different TLR2 ligands, the distinct physiologic response to LTA versus the other TLR2 ligands argues that the kinetic differences in pathway activation may indeed have profound effects on the cellular responses to these different ligands. Therefore, the results presented herein may herald the identification of ligand-specific intricacies, which are reminiscent of the TLR4 signaling paradigm, within the biology of TLR2 signaling pathways.
